# Safety and feasibility of a Dalcroze eurhythmics and a simple home exercise program among older adults with mild cognitive impairment (MCI) or mild dementia: the MOVE for your MIND pilot trial

**DOI:** 10.1186/s40814-020-00645-7

**Published:** 2020-07-15

**Authors:** Melanie Fischbacher, Patricia Orializ Chocano-Bedoya, Ursina Meyer, Irene Bopp, Michèle Mattle, Reto Werner Kressig, Andreas Egli, Heike Annette Bischoff-Ferrari

**Affiliations:** 1 Department of Geriatrics, University Hospital Zurich, Rämistrasse 100, 8091 Zürich, Switzerland; 2 Center on Aging and Mobility, University Hospital Zurich, Waid City Hospital, and University of Zurich, Tièchestrasse 99, 8037 Zürich, Switzerland; 3 School of Allied Health Sciences, Griffith University, Gold Coast Campus, Gold Coast, QLD 4222 Australia; 4 University Clinic for Acute Geriatric Care, Waid City Hospital, Tièchestrasse 99, 8037 Zürich, Switzerland; 5 Felix Platter-Hospital, University Center for Medicine of Aging, Burgfelderstrasse 101, Postfach, 4002 Basel, Switzerland

**Keywords:** Dementia, MCI, Eurhythmics, Exercise, Older adults

## Abstract

**Background:**

Falls represent a major health problem for older adults with cognitive impairment, and the effects of exercise for fall reduction are understudied in this population. This pilot randomized controlled trial evaluated the feasibility, safety, and exploratory effectiveness of a Dalcroze eurhythmics program and a home exercise program designed for fall prevention in older adults with mild cognitive impairment (MCI) or early dementia.

**Methods:**

For this three-arm, single-blind, 12-month randomized controlled pilot trial, we recruited community-dwelling women and men age 65 years and older with MCI or early dementia through participating memory clinics in Zurich, Switzerland. Participants were randomly assigned to a Dalcroze eurhythmics group program, a simple home exercise program (SHEP), or a non-exercise control group. All participants received 800 IU of vitamin D_3_ per day. The main objective of the study was to test the feasibility of recruitment and safety of the interventions. Additional outcomes included fall rate, gait performance, and cognitive function.

**Results:**

Over 12 months, 221 older adults were contacted and 159 (72%) were screened via telephone. Following screening, 12% (19/159) met the inclusion criteria and were willing to participate. One participant withdrew at the end of the baseline visit and 18 were randomized to Dalcroze eurhythmics (*n* = 7), SHEP (*n* = 5), or control (*n* = 6). Adherence was similarly low in the Dalcroze eurhythmics group (56%) and in the SHEP group (62%; *p* = 0.82). Regarding safety and pilot clinical endpoints, there were no differences between groups.

**Conclusion:**

The MOVE for your MIND pilot study showed that recruitment of older adults with MCI or early dementia for long-term exercise interventions is challenging. While there were no safety concerns, adherence to both exercise programs was low.

**Trial registration:**

ClinicalTrials.gov, NCT02279316. Registered on 31 October 2014

## Background

Falls are highly prevalent in older adults with every third person over the age of 65 years experiencing at least one fall per year [1, 2]. Falls also constitute a significant health burden as 10–20% of all falls have serious consequences such as fractures, hospitalization, or death [3, 4]. Falls are multifactorial, and major risk factors include older age, gait and balance impairment, and reduced cognitive function [4, 5]. Regarding the latter, older adults with mild cognitive impairment (MCI) or dementia have a twofold higher risk of falling and are twice as likely to experience severe consequences such as fractures and loss of autonomy compared to cognitively healthy peers [1, 6].

Exercise is among the most effective strategies for fall prevention, independent of whether it is performed in a group setting or at home [7, 8]. Notably, both settings have been demonstrated to be safe and feasible in older adults with cognitive impairment or dementia [9–11]. However, results from meta-analyses on the effect of exercise on fall reduction among older adults with impaired cognitive function are inconclusive, indicating that the beneficial effect of certain exercise programs might not translate to this population [12–14].

Music-based exercise programs stimulate both motor [15] and cognitive functions [16] and may be particularly beneficial for cognitively impaired older adults [17]. Dalcroze eurhythmics training is a music-based multi-task training which appeals to balance and gait functions, but also to concentration, alertness, and executive functions [18]. In a previous randomized controlled trial, once weekly participation in a Dalcroze eurhythmics program reduced fall rate by 54% and significantly improved gait, balance, and executive functions among cognitively healthy, community-dwelling older adults [19, 20]. Attending a group exercise intervention outside the home, such as Dalcroze eurhythmics, requires a certain level of organizational skills and might therefore not be feasible for a significant portion of older adults at early stages of cognitive impairment.

In order to establish feasibility (based on recruitment rates, adherence, and dropout rates) and to collect pilot data on the clinical effectiveness of two different exercise strategies for cognitively impaired older adults, a Dalcroze eurhythmics program and a simple home exercise program (SHEP) were compared to a non-exercise control group among older adults with MCI or early dementia. The SHEP has been validated in the Zurich hip fracture trial and reduced the rate of falls significantly by 25% in older adults with acute hip fracture with a mean age of 84 years [21, 22].

## Methods

The reporting of this study follows the CONSORT statement for randomized pilot and feasibility trials [23].

### Study design

The MOVE for your MIND pilot trial is a 12-month, single-blind randomized controlled trial, conducted between August 2014 and September 2016. Participants were randomly allocated to either *Dalcroze eurhythmics* classes (1 × 60 min/week), the *SHEP* (3 × 30 min/week), or the *control* group in a parallel group trial design. All participants received 800 IU of vitamin D_3_ per day to maintain standard of care. The primary endpoints were feasibility assessed by recruitment rate, adherence, and safety. Secondary endpoints were rate of falls, gait performance, and cognitive function.

### Setting and participants

Participants were recruited from three memory clinics in Zurich cooperating in the study (City Hospital Waid, Entlisberg, and Rehalp). Recruitment strategies involved mailing lists from the three memory clinics as well as referrals through practicing physicians at the memory clinics. All memory clinics are members of the Swiss Memory Clinics Association and therefore apply the same diagnostic testing which includes physical examination, extensive neuropsychological testing, pathology (blood markers), and magnetic resonance imaging. The diagnosis is made in an interdisciplinary team based on the results of the testing.

The target sample size was 60 participants; however, only 18 participants could be enrolled. This study was therefore defined as an *a posteriori* pilot trial in order to address feasibility endpoints for future studies.

Memory clinic patients who had been diagnosed with MCI or early dementia 12 months prior to the study start or during the recruitment period were informed about the study by their attending physician who also made the decision whether a patient is judicious based on the comprehensive diagnostic testing. Participants were only eligible if confirmed to be able to give informed consent by their attending physician at the memory clinic. Other study eligibility criteria included age 65 years or older and being sufficiently mobile to come to the study center and comply with the intervention. Prospective participants who expressed interest in the study underwent a preliminary eligibility screening over the phone. Medical records from the memory clinics were also considered during that initial screening process. If participants were eligible and willing to participate after the pre-screening, participants were invited for the screening and baseline visit. At the beginning of the initial visit at the study center, a study doctor performed the final screening interview, which involved the same questions as the pre-screening interview, to confirm eligibility of the participant.

After we recruited the first eleven participants into the trial, we changed one eligibility criterion and started to recruit an informant (e.g., partner, caregiver) for each participant to ensure reliability of fall reports and to improve adherence to the exercise programs. Key exclusion criteria were diseases associated with an increased risk of falling (e.g., Parkinson’s disease, polyneuropathy, vertigo) or other serious illnesses (e.g., cancer, kidney failure, coronary heart disease).

The study protocol was approved by the Cantonal Ethics Committee of Zurich (2014-0110). Written informed consent was obtained from all study participants before any study-related procedure was conducted. Participant enrollment, all examinations, and data collection took place at the Centre on Aging and Mobility at the University of Zurich.

### Randomization and blinding

Randomization was performed in blocks of six and stratified by the diagnosis of cognitive impairment (MCI vs. dementia) and history of falls during the last 12 months (any vs. none). A random allocation number for each participant was computer-generated by the trial software.

All study staff members were blinded to treatment allocation with the exception of two study nurses involved in the recruitment and organization of the Dalcroze eurhythmics classes and the physiotherapist, who completed the randomization and introduced participants to the treatment allocation, performed instruction of the SHEP, and assessed adherence to the exercise program.

### Interventions

The Dalcroze eurhythmics classes followed a protocol which was successfully used in a previous study [19]. Classes took place once weekly for 60 min and were taught by an experienced instructor. Classes were held every week over the 12-month intervention period (52 weeks) except for public holidays and a short break over Christmas and summer holidays, amounting to a total of approximately 47 sessions per year. The exercises included different courses of motion, sometimes in combination with the handling of objects (e.g., a ball or claves), performed to the rhythm of piano music. The complexity of the exercises gradually increased during a session, starting with single tasks and then combining them into multi-task exercises. Adherence to the Dalcroze eurhythmics sessions was recorded by the instructor.

The SHEP was tested in a prior trial among acute hip fracture patients and included five components: sit-to-stand, single leg stance (balance component), stair climbing (functional mobility), pull back, and external shoulder rotation against elastic resistance [21]. It was instructed by a physiotherapist at the baseline visit and adapted to individual needs if necessary. Participants were provided a booklet with a detailed description and illustration of the program with the recommendation to do the exercises three times a week. We also provided a training diary to record adherence to the exercise program. Participants were instructed to perform the exercises three times a week for the entire 12 months.

Based on pre-defined criteria, participants were considered adherent if they attended 80% of the Dalcroze eurhythmics classes or if they performed the SHEP at least once a week on average [21].

### Outcome measures

Feasibility of recruitment and the exercise interventions was assessed by measuring recruitment rate, exercise adherence, and dropout rates. Adverse events were recorded to evaluate safety of the trial interventions and testing procedures.

The study included three clinical visits (baseline and 6 and 12 months) which involved physical examination, medical history, gait analyses, and functional and cognitive tests. Between clinical visits, participants were contacted bi-monthly to record adverse events, adherence to the SHEP, and falls. Falls were defined as “unintentionally coming to rest on the ground, floor, or other lower level.” Coming to rest against furniture or a wall was not considered a fall [24]. Incident falls were assessed with a self-reported diary, at the 6- and 12-month visits and at the bi-monthly phone calls.

Gait speed and variability of step length and step time were assessed using the GAITRite® gait analysis system, a pressure-sensitive walkway of 792 cm length (Platinum CIR Systems, PA, USA). Participants completed three single-task walks: walking at preferred speed (referred to as “normal” gait speed), slow speed, and fast speed. Then, they performed the dual-task walks: walking at normal speed while serially subtracting two, starting from a pre-defined number (working memory dual-task), and walking while naming animals (semantic memory dual-task). Coefficients of variation (CV) were calculated for step length and step time parameters (CV = [SD/mean] × 100).

Cognitive function was assessed using the Montreal Cognitive Assessment (MoCA). The MoCA test consists of 30 questions evaluating different cognitive abilities. The score can range from 0 to 30 points, and a score of ≥ 26 is considered normal. In the validation study, the average score for patients with MCI was 22.1 and 16.2 for people with Alzheimer’s disease [25].

### Statistical analyses

Baseline characteristics were compared by treatment group. We used Fischer exact tests for categorical variables due to small cell counts. For continuous variables that had a normal distribution, we used ANOVA *F* tests, and for those with high skewness or kurtosis when stratified by treatment, we used Kruskal-Wallis non-parametric tests.

Recruitment rate was calculated as the proportion of participants who were successfully enrolled in the trial of the total number screened for eligibility. Adherence rate was defined as the percentage of exercise sessions completed of the maximum number of sessions they could have attended during the 12-month intervention period (100%).

We compared the change of single- and dual-task gait speed and MoCA over time between the 3 study groups using a repeated-measures linear mixed model. All analyses were conducted in SAS 9.4.

## Results

### Recruitment

**Fig. 1:**
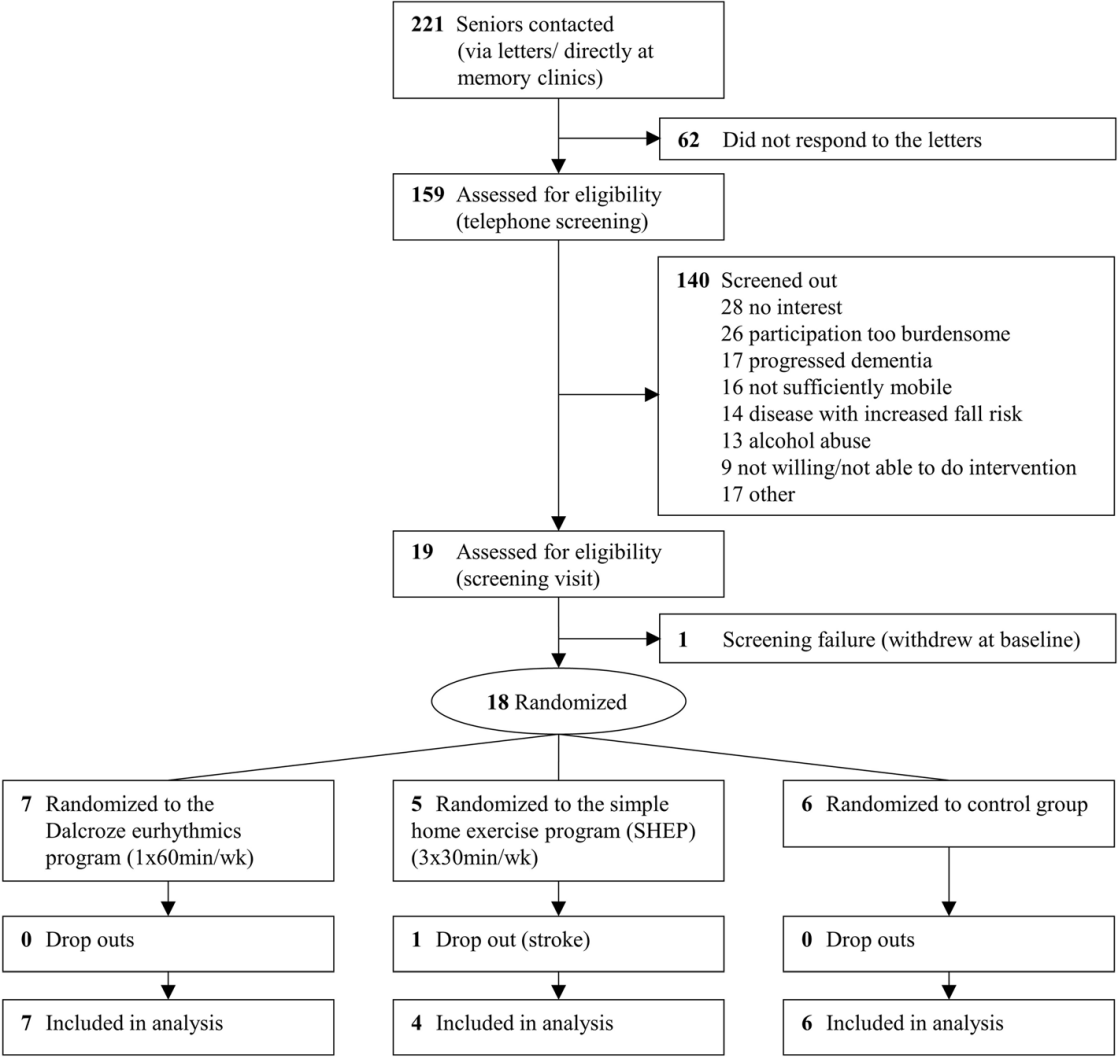
CONSORT diagram of participant flow

Of 221 older adults contacted with possible MCI or early dementia, 18 were enrolled (Fig. 1) and randomized to Dalcroze eurhythmics classes (*n* = 7), SHEP (*n* = 5), or control (*n* = 6). At baseline, nine participants (50%) reported a fall in the 12 months prior to enrollment. Baseline characteristics are shown in Table 1. The mean age of the study participants was 76.4 ± 4.5 years at baseline, and the mean MoCA score was 20.6 ± 2.8. None of the baseline characteristics differed significantly between treatment groups. However, participants in the Dalcroze group had slightly lower gait speed for the dual-task walks. One study participant dropped out of the study after 150 days of follow-up because of a hemorrhagic insult.

**Table 1:** Baseline characteristics by treatment group

Variable	Dalcroze (*n* = 7)	SHEP (*n* = 5)	Control (*n* = 6)
Age, years	75.9 (5.1)	73.8 (3.6)	76.7 (4.8)
Female gender, *n* (%)	4 (57.1)	3 (60.0)	4 (66.7)
Fall history last 12 months, *n* (%)	4 (57.1)	2 (40.0)	3 (50.0)
Cognitive function			
Diagnosis of MCI, *n* (%)	3 (42.9)	2 (40.0)	1 (16.7)
Diagnosis of mild dementia, *n* (%)	4 (57.1)	3 (60.0)	5 (83.3)
MoCA score (max. 30)	19.6 (2.9)	22.6 (3.0)	20.0 (1.9)
Gait speed, cm/s			
Single task	103.3 (20.8)	104.4 (31.6)	112.0 (13.8)
Dual-task working memory	90.2 (19.0)	114.5 (19.3)	88.3 (17.2)
Dual-task semantic memory	94.5 (73.4–106.5)	128.5 (78.9–149.1)	94.5 (73.4–106.5)

### Adherence to the study interventions

Adherence to both exercise interventions was low. In the SHEP group, the average adherence was 62% with three out of five participants being compliant (trained at least once per week on average). In the Dalcroze group, only two out of seven participants were compliant (attended ≥ 80% of the sessions) and mean adherence was 56%. The reasons for non-adherence to the exercise interventions were forgetting about the program, interference with other activities or therapies, lack of motivation to leave home, or illness.

### Study outcomes

Given the challenges of recruitment and small sample size, we cannot draw any conclusions regarding the effectiveness of our interventions based on this pilot study.

#### Rate of falling

Five falls were reported during the 12-month follow-up period, one fall occurred in the control group and two falls in each intervention group.

#### Gait performance

Analyses for single-task normal gait speed did not significantly differ between groups, with a non-significant improvement from baseline to 12 months by 6.5 cm/s (mean change = + 6.5 cm/s; 95% CI − 18.3, 31.4) for the SHEP group, a decline by 10 cm/s (mean change = − 10.0 cm/s; 95% CI − 28.2, 8.3) for the Dalcroze group, and a decrease of 10.3 cm/s (mean change = − 10.3 cm/s; 95% CI − 31.2, 10.6) for the control group.

Similarly, there was no difference between groups in gait speed during working memory dual-task from baseline to 12 months, with a small decrease of 0.9 cm/s in the Dalcroze group in contrast to a decrease of 39.5 cm/s in the SHEP group and 26.1 cm/s in the control group. Results for single- and dual-task gait speed are summarized in Table 2.

**Table 2:** Changes over time for gait speed and MoCA

		Dalcroze (*n* = 7)	SHEP (*n* = 4)	Control (*n* = 6)
**Change in single-task gait speed**				
Mean gait speed at baseline (95% CI)		103.3 (84.1, 122.6)	104.4 (65.2, 143.7)	112.0 (97.5, 126.5)
Mean change (95% CI) at 6 months		− 11.4 (− 33.4, 10.6)	15.4 (− 14.3, 45.0)	5.0 (− 19.8, 29.8)
Mean change (95% CI) at 12 months		− 10.0 (− 28.2, 8.3)	6.5 (− 18.3, 31.4)	− 10.3 (− 31.2, 10.6)
**Change in dual-task working memory**				
Mean gait speed at baseline (95% CI)		90.2 (72.7, 107.8)	114.5 (90.5, 138.4)	88.3 (70.2, 106.4)
Mean change (95% CI) at 6 months		− 0.1 (− 32.0, 31.8)	− 45.5 (− 60.0, − 19.1)	− 15.3 (− 50.1, 19.4)
Mean change (95% CI) at 12 months		− 0.9 (− 14.8, 13.1)	− 39.5 (− 87.7, − 3.3)	-26.1 (− 41.4, − 10.8)
**Change in dual-task semantic memory**				
Mean gait speed at baseline (95% CI)		79.1 (61.8, 96.3)	114.0 (78.9, 149.1)	89.9 (73.4, 106.5)
Mean change (95% CI) at 6 months		− 32.4 (− 71.3, 6.5)	− 20.4 (− 73.0, 32.2)	− 26.3 (− 64.1, 11.6)
Mean change (95% CI) at 12 months		-16.4 (− 46.4, 13.6)	− 26.7 (− 69.7, 16.3)	− 22.2 (− 50.2, 5.8)
**Changes in MoCA**				
Mean MoCA Score at baseline (95% CI)		19.6 (16.9, 22.2)	22.6 (18.9, 26.3)	20.0 (18.0, 21.2)
Mean change (95% CI) at 6 months		0.3 (− 2.0, 2.6)	− 3.7 (− 6.9, − 0.5)	− 0.6 (− 0.3, 1.9)
Mean change (95% CI) at 12 months		− 1.0 (− 2.5, 0.5)	− 1.7 (− 3.9, 0.4)	0.6 (− 1.0, 2.2)

#### Cognitive function

For the change in MoCA from baseline to 12 months, there was no difference between groups, with a non-significant decline for both the Dalcroze (− 1.0 points) and the SHEP (− 1.7 points) groups and a non-significant improvement in the control group (a 0.6-point increase) (Table 2).

### Safety

Five serious adverse events were reported: one in the control group (cerebrovascular insult), one in the SHEP group (hemorrhagic stroke), and three in the Dalcroze group (partial bowel obstruction, theophylline intoxication, retinal detachment). None of the events was related to the interventions, and all participants continued their participation except for the one who dropped out.

## Discussion

The aim of the MOVE for your MIND pilot trial was to evaluate the feasibility and safety of two exercise strategies, a Dalcroze eurhythmics program or a simple home exercise program (SHEP), among older adults with MCI or early dementia. While the results of our study suggest safety of the interventions, they do not confirm feasibility with regard to recruitment and adherence in this target population.

While the desired sample size was 60 participants, only 18 of 220 contacted older adults from 3 memory clinics could be enrolled in the pilot trial, reflecting a recruitment rate of 8.1%. Recruitment strategies included mailing lists of the recruiting memory clinics of potentially eligible participants and referrals through practicing physicians at the memory clinics. Recruitment challenges included comorbidities such as mobility impairment, logistical barriers (e.g., traveling to the study center), and unwillingness to participate in a research project.

Although short screening tools like MMSE [26] and MoCA have a relatively high sensitivity and specificity [25], they are not precise enough for diagnostic purposes, especially in early stages of dementia [27]. Our pilot trial therefore tested the feasibility of recruitment through memory clinics, and not newspapers or general mailing lists. Participant recruitment is a well-known barrier to dementia research, and exploring the reasons and ways to overcome these challenges is a priority [28, 29]. Similar to our study, a lack of interest and research awareness and high number of comorbidities were the major factors that challenged recruitment of older adults with dementia in previous studies [28]. Prior trials of dementia prevention strategies have recruited patients from a combination of sources including memory clinics from several different cities and dementia registers and networks [9–11]. Compared to our study, recruitment in those studies seemed more successful. However, recruitment through dementia registers is not always possible due to privacy reasons and limited possibilities to access the registry. Another explanation for the higher recruitment rates in previous studies might be that they had multiple testing and training centers [9, 11]. The fact that all our participants had to be willing and able to attend our study center once weekly in case they got randomized to Dalcroze eurhythmics training might have been a barrier to successful recruitment.

Among the few participants recruited in our pilot trial, retention rate was high with only one participant (5.5%) dropping out of the study. However, adherence to the study interventions was low for both the Dalcroze group (56% adherence) and the SHEP group (62% adherence). The main reason for non-adherence was forgetting about the program or lack of motivation to leave home. We think that the duration of each session for both programs was adequate although a 60-min Dalcroze session might seem long for patients with cognitive impairment. The 1-h duration of the Dalcroze sessions was chosen based on a previous trial [19] and the previous experience of our investigator team and the eurhythmics instructors with Dalcroze classes in this population. Also, lessons were targeted to the capabilities of each participant, and they had the possibility to sit down for the exercises when needed.

This study has several strengths. First, the study was of high methodological quality regarding the comprehensive testing applied at the memory clinics to diagnose MCI or mild dementia, randomization, blinding, testing, and analysis procedures. Second, the tests and questionnaires used in this study have been validated in the senior population and previously applied in older adults with cognitive impairment. Third, all participants have been diagnosed with the same, high standard diagnostic setting before they were enrolled. Nevertheless, the main limitation of this study is that the high standard of diagnostic and enrollment criteria made the recruitment very challenging and resulted in a small sample size. Also, our results are not applicable to older adults who are either cognitively healthy or affected by more severe stages of dementia.

The challenges of recruitment and adherence led to the conclusion that a trial testing a home versus a group exercise program for patients with MCI or early dementia is not feasible. Participants at an earlier stage in the continuum of cognitive decline may be more likely to be enrolled and motivated for preventive exercise interventions. In fact, an ideal target population may be older adults with subjective cognitive decline (SCD). SCD is a preclinical state of dementia during which affected individuals already experience deterioration in their cognitive function; however, the changes are not objectively measurable [30]. SCD is an important risk factor for objective cognitive impairment such as MCI and dementia [30], and the period between individuals first experience SCD until they get diagnosed with MCI or dementia could be considered a window of opportunity for early interventions to prevent cognitive decline and falls. This is supported by recent dementia research guidelines which recommend to include participants with earlier diagnostic disease stages before the full picture of dementia is reached [31].

## Conclusion

Given the challenges of recruitment and adherence among community-dwelling patients with MCI or early dementia, a more effective strategy for RCTs on exercise interventions and cognitive health may be a focus on older adults at an earlier state of cognitive decline. Consequently, the definitive trial building on this pilot study started recruitment in January 2018 (clinicaltrials.gov ID: NCT03384602) targeting older adults with SCD.

## Data Availability

The datasets used and/or analyzed during the current study are not publicly available due to patient privacy but are available from the corresponding author on reasonable request.

## Abbreviations

MCI: Mild cognitive impairment
MMSE: Mini Mental State Examination
MOCA: Montreal Cognitive Assessment
RCT: Randomized controlled trial
SCD: Subjective cognitive decline
SHEP: Simple home exercise program
